# Different Influences of Biotinylation and PEGylation on Cationic and Anionic Proteins for Spheroid Penetration and Intracellular Uptake to Cancer Cells

**DOI:** 10.4014/jmb.2207.07058

**Published:** 2022-08-29

**Authors:** Won Ho Jung, Gayeon You, Hyejung Mok

**Affiliations:** Department of Bioscience and Biotechnology, Konkuk University, Seoul 05029, Republic of Korea

**Keywords:** PEGylation, biotinylation, cationic protein, anionic protein, spheroid penetration, intracellular uptake

## Abstract

To better understand the effects of PEGylation and biotinylation on the delivery efficiency of proteins, the cationic protein lysozyme (LZ) and anionic protein bovine serum albumin (BSA) were chemically conjugated with poly(ethylene glycol) (PEG) and biotin-PEG to primary amine groups of proteins using N-hydroxysuccinimide reactions. Four types of protein conjugates were successfully prepared: PEGylated LZ (PEG-LZ), PEGylated BSA (PEG-BSA), biotin-PEG-conjugated LZ (Bio-PEG-LZ), and biotin-PEG-conjugated BSA (Bio-PEG-BSA). PEG-LZ and Bio-PEG-LZ exhibited a lower intracellular uptake than that of LZ in A549 human lung cancer cells (in a two-dimensional culture). However, Bio-PEG-BSA showed significantly improved intracellular delivery as compared to that of PEG-BSA and BSA, probably because of favorable interactions with cells via biotin receptors. For A549/fibroblast coculture spheroids, PEG-LZ and PEG-BSA exhibited significantly decreased tissue penetration as compared with that of unmodified proteins. However, Bio-PEG-BSA showed tissue penetration comparable to that of unmodified BSA. In addition, citraconlyated LZ (Cit-LZ) showed reduced spheroid penetration as compared to that of LZ, probably owing to a decrease in protein charge. Taken together, chemical conjugation of targeting ligands-PEG to anionic proteins could be a promising strategy to improve intracellular delivery and in vivo activity, whereas modifications of cationic proteins should be more delicately designed.

## Introduction

Biological drugs, such as genes, proteins, and cells, have significantly broadened the therapeutic scope for the treatment of incurable diseases such as cancers and brain diseases [1-3]. Most biological drugs are administered via intravenous injection owing to their intrinsic physicochemical properties such as high molecular weight, charged and hydrophilic surfaces, and enzymatic instability [4]. After the systemic administration of anticancer drugs in vivo, several delivery hurdles, such as serum half-life, extravasation, and tissue penetration, should be overcome for sufficient accumulation in target tumor tissues and subsequent therapeutic activity. After biological drugs are located near tumor tissues via extravasation, the degree of penetration into the interior of the tumor may be essential for antitumor effects [5]. Owing to insignificant tissue penetration and negligible intracellular uptake of unmodified therapeutic proteins, most developed protein drugs have been designed to target the exterior regions of transmembrane proteins. However, biological drugs active in intracellular regions, such as the CRISPR-associated protein 9, zinc finger nuclease, and recombinase, should be delivered into the cytoplasm and other intracellular organelles [1, 6, 7]. Various polymer-based and lipid-based carriers have been investigated to improve the intracellular delivery of protein drugs [6, 7].

Poly(ethylene) glycol conjugation (PEGylation), a representative and translated technique of protein modification, has been considered a promising strategy to improve the intrinsic limitations of therapeutic proteins such as insignificant enzymatic stability and short in vivo half-life [8, 9]. Accordingly, the effects of PEGylation on the adsorption of plasma proteins, mucus penetration, circulation time, and blood clearance have been extensively investigated [10]. In previous studies, PEGylated tumor necrosis factor-alpha and PEGylated interferon showed a 10–30-fold longer half-life than that of the unmodified proteins. However, to the best of our knowledge, the effects of PEGylation on different types of proteins with different physicochemical properties, such as molecular weight and charge, for tissue penetration and intracellular uptake have not been examined in detail. In addition, conjugation of targeting ligands could be a popular strategy to improve the intracellular delivery of proteins. Several promising ligands, such as folate, biotin, and arginylglycylaspartic acid (RGD) peptides, have been harnessed to modify functional proteins for efficient intracellular delivery to cancer cells [11-13]. In particular, biotin is a vitamin B7, which is an essential component for cell growth and proliferation and has been popularly examined as a targeting ligand for the cancer-specific delivery of anticancer drugs [14, 15]. However, the effects of cationic and anionic protein modification with targeting ligands on cytosolic delivery have not been investigated.

To better understand the roles of chemical conjugations with PEG and targeting ligands to therapeutic proteins in terms of the delivery efficiency, effects of PEGylation and biotinylation to different types of proteins on tissue penetration and intracellular uptake should be comparatively examined. In this study, we prepared four types of protein conjugates using the cationic protein lysozyme (LZ) and anionic protein bovine serum albumin (BSA). N-hydroxysuccinimide (NHS)-activated PEG and biotin-PEG were chemically reacted with the primary amine groups of LZ and BSA in different protein/polymer molar ratios. PEGylated LZ (PEG-LZ), PEGylated BSA (PEG-BSA), biotin-PEG conjugated LZ (Bio-PEG-LZ), and biotin-PEG conjugated BSA (Bio-PEG-BSA) were analyzed using polyacrylamide gel electrophoresis and matrix-assisted laser desorption ionization time-of-flight mass spectrometry (MALDI-TOF MS). After fluorescence labeled protein conjugates were treated to the A549 (human lung cancer cell line) cells in two-dimensional (2D) cell culture conditions. the extent of internalized proteins was quantitatively assessed using a fluorospectrophotometer. In addition, fluorescence-labeled protein conjugates were treated with three-dimensional (3D) A549/fibroblast coculture spheroids for 8 h to assess the degree of spheroid penetration using confocal microscopy. To examine the effects of protein charge on tissue penetration, the degree of penetration of LZ and citraconlyated LZ (Cit-LZ) were also comparatively investigated for 3D coculture spheroids.

## Materials and Methods

### Materials

BSA, LZ from chicken egg whites, phosphate-buffered saline (PBS), dimethyl sulfoxide (DMSO), Triton X-100, formaldehyde solution, fluorescamine, fluorescein isothiocyanate isomer I (FITC), poly(ethylene glycol) (N-hydroxysuccinimide 5-pentanoate) ether 2-(biotinylamino)ethane (NHS-activated biotin-PEG, MW 3800), and citraconic anhydride were purchased from Sigma-Aldrich (USA). Dulbecco’s modified Eagle’s medium (DMEM), Roswell Park Memorial Institute (RPMI) 1640 medium, penicillin/streptomycin (P/S), trypsin-EDTA (T/E), and fetal bovine serum (FBS) were purchased from Gibco BRL (USA). The micro-BCA protein assay kit, GelCode Blue Stain Reagent, and Coomassie Protein Assay Reagent were obtained from Thermo Scientific, USA). Methoxy polyethylene glycol succinimidyl succinate (NHS-activated PEG, MW 5000) was purchased from SunBio Co.(Republic of Korea).

### Preparation and Characterization of Proteins Conjugates

To prepare FITC-conjugated proteins, LZ (10 mg) and BSA (40 mg) in a sodium carbonate-bicarbonate buffer (pH 9.0) were reacted with FITC for 3.5 h at 4°C overnight, respectively. After the reaction, the solvent and byproducts were removed using dialysis (MWCO 3.5 kDa) against deionized water (DW) at 4°C for 1 d. To prepare FITC-LZ-PEG, FITC-LZ (2.2 mg) in PBS (pH 7.4) was reacted with NHS-activated PEG (125 mg/ml) at different NHS-activated PEG/LZ molar ratios (1 and 5) for PEG-LZ 1 and PEG-LZ 5 by stirring for 2 h at 4°C. After the reaction, the solvent and byproducts were removed using dialysis against DW at 4°C for 1 d. To prepare FITC-BSA-PEG, FITC-BSA (10 mg) in a PBS solution (pH 7.4) was reacted with NHS-activated PEG (125 mg/ml) at various NHS-activated PEG/BSA molar ratios (1, 5, and 10) by stirring for 2 h at 4°C for PEG-BSA 1, PEG-BSA 5, and PEG-BSA 10, respectively. After the reaction, the solvent and byproducts were removed using dialysis (MWCO 10 kDa) against DW at 4°C for 1 d. FITC-LZ-PEG (10 μg) and FITC-BSA-PEG (6 μg) were loaded onto 15 and 10% sodium dodecyl sulfate-polyacrylamide gel (SDS-PAGE) at 150 V for 60 min and 200 V for 40 min, respectively. After gel electrophoresis, the migration of samples was visualized with an IVIS instrument (Caliper Life Sciences Lumina II, USA) at excitation and emission wavelengths of 480 and 520 nm, respectively. The synthesized Bio-PEG-LZ and Bio-PEG-BSA were also analyzed using MALDI-TOF MS (AB SCIEX TOF/TOF 5800 system, USA) compared to unmodified LZ and BSA. A solution of α-cryano-4-hydroxycinnamic acid (sinapinic acid, 10 mg/ml in 0.1% TFA/30% acetonitrile) was used as a matrix for the MS analysis.

### Cellular Uptake of Protein Conjugates

A549 cells were maintained in the RPMI-1640 supplemented with 10% FBS, 100 U/ml penicillin, and 100 μg/ml streptomycin at 37°C in a humidified atmosphere with 5% CO_2_. A549 cells were plated at a density of 4 × 10^5^ cells per well in a 6-well plate 24 h before treatment. Cells were treated with FITC-LZ-PEG conjugates (NHS-activated PEG/LZ molar ratios of 0, 1, and 5) and FITC-BSA-PEG conjugates (NHS-activated PEG/BSA molar ratios of 0, 5, and 10) at a final FITC concentration of 2.2 μg/ml for 4 h in a serum-containing media (10%). After washing with PBS solution, cells were lysed using a lysis buffer (1% Triton X-100 in PBS solution). After centrifugation at 13,000 rpm for 10 min, the supernatant was analyzed using a spectrofluorophotometer at the excitation and emission wavelengths of 480 and 520 nm, respectively.

### Preparation of Coculture Spheroids

A549 cells and human fibroblasts (CCD-1059-SK cells) were maintained in RPMI-1640 and DMEM supplemented with 10% FBS, 100 U/ml penicillin, and 100 μg/ml streptomycin, respectively, at 37°C. For coculture 3D spheroids, A549 (1 × 10^4^ cells) and fibroblasts (1 × 10^3^ cells) cells were seeded in an ultra-low attachment 96-well plate (Corning, USA) at 37°C in a humidified atmosphere of 5% CO_2_ for 2 d. A549 spheroids and A549/fibroblast coculture spheroids were fixed with 2.5% glutaraldehyde and 1% osmium tetroxide for 1 h at 4°C and dehydrated with ethanol (25, 50, 75, and 100% serial dehydration). The size and morphology of A549 spheroids and A549/fibroblasts coculture spheroids were observed using ultra-high resolution scanning electron microscope (SEM; Hitachi High-Technologies Corp., Japan). The diameters of A549 spheroids and A549/fibroblasts coculture spheroids were quantitatively analyzed using ImageJ software.

### Spheroid Penetration Assay

For the penetration assay of LZ and BSA, A549/fibroblasts coculture spheroids were treated with FITC-BSA-PEG conjugates (NHS-activated PEG/BSA molar ratios of 0, 5, and 10) and FITC-LZ-PEG conjugates (NHS-activated PEG/LZ molar ratios of 0, 1, and 5) at a final FITC concentration of 8 μg/ml for 8 h in 10% serum-containing media. After incubation, the spheroids were washed twice with PBS solution, and the samples were incubated with 3.7% paraformaldehyde in PBS for 30 min at RT. Finally, the spheroids were mounted in a PBS solution with 0.5% agarose and visualized by confocal microscopy (Carl Zeiss, Germany). Moreover, a z-stacking analysis from the surface into the spheroids at 10 fixed slices was conducted. The mean fluorescence intensity of FITC in each z-stack slice was quantitatively analyzed using Zen version 3 Blue Edition software.

### Preparation and Spheroid Penetration Assay of Cit-LZ

To prepare Cit-LZ, amine groups on LZ (2.5 mg) in the PBS solution were reacted with citraconic anhydride at various citraconic anhydride/amine groups in LZ molar ratios (0, 0.1, 0.5, and 1) by stirring for 2 h at 4°C. After the reaction, the solvent and unreacted citraconic anhydride were removed using dialysis (MWCO 3.5 kDa) against DW at 4°C for 1 d. The amount of remaining amine groups in the resulting conjugates was quantified by fluorescamine assay according to the manufacturer’s protocol. The resulting conjugates, FITC-LZ and cit-FITC-LZ were loaded into 15% non-denaturing and denaturing polyacrylamide gels. After gel electrophoresis, the location of each sample was visualized using an IVIS instrument at excitation and emission wavelengths of 480 and 520 nm, respectively.

For the penetration assay of citraconylated conjugates, A549/fibroblasts coculture spheroid cells were treated with FITC-LZ and cit-FITC-LZ for 8 h in 10% serum-containing media at a final FITC concentration of 2.5 μg/ml. After washing the spheroids twice with PBS solution, the samples were incubated with 3.7% paraformaldehyde in a PBS solution for 30 min at RT. Finally, the spheroids were mounted in a PBS solution with 0.5% agarose and visualized by confocal microscopy (Carl Zeiss). Subsequently, a z-stacking analysis from the surface into the spheroids at 10 fixed slices was conducted. The mean FITC intensity in each z-stack slice was quantitatively analyzed using Zen version 3 Blue Edition software.

## Results and Discussion

### Preparation and Characterization of Proteins Conjugates

**Fig. 1:**
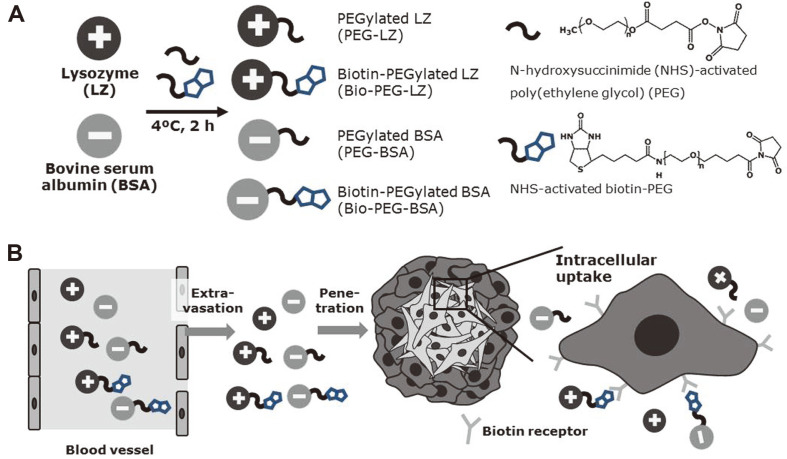
Influences of biotinylation and PEGylation on cationic and anionic proteins. (**A**) Synthetic scheme for the preparation of PEG-LZ, Bio-PEG-LZ, PEG-BSA, and Bio-PEG-BSA. (**B**) Schematic illustration of tumor penetration and uptake into cancer cells after extravasation of PEGylated proteins.

In this study, two types of model proteins, LZ (isoelectric point: 11) and BSA (isoelectric point: 4.8-5.6), were used as cationic and anionic protein models, respectively [16, 17]. Biotin was selected as the targeting ligand because of the high expression level of biotin receptors in a wide range of cancer cells (MCF-7, HeLa, and A549 cells) [18]. We prepared four types of PEGylated and biotinylated proteins, PEG-LZ, Bio-PEG-LZ, PEG-BSA, and Bio-PEG-BSA, using chemical conjugation methods. The primary amine groups of LZ and BSA reacted with NHS-activated PEG and NHS-activated biotin-PEG, respectively (Fig. 1A). Fig. 1B shows the form of the protein conjugates after in vivo systemic administration. After blood circulation and subsequent extravasation of protein conjugates, they penetrated the interior of tumor tissues composed of diverse types of cells (cancer cells, fibroblasts, macrophages, dendritic cells, and T-cells) with high pressure and low pH for anticancer effects [19]. In this study, coculture spheroids composed of A549 and human fibroblasts were prepared to mimic 3D tumor tissues, according to a previous study [20]. After the localization of protein conjugates near cancer cells, cytosolic therapeutic proteins should be taken up into cancer cells for intracellular processing, for example, interaction with cytoplasmic molecules and regulation of intracellular pathways. Cationic proteins favorably interacted with the plasma membrane as compared with anionic proteins, owing to the negative charge of anionic phospholipids such as phosphatidylserine and phosphatidic acid on the plasma membrane [21, 22]. Biotinylated proteins could interact with biotin receptors, which could subsequently allow receptor-mediated endocytosis for the internalization of proteins. PEGylation enhanced blood circulation, however, it could also interfere with the interactions of PEGylated proteins with the plasma membrane. In this study, we hypothesized that the effects of PEGylation and targeting ligand conjugation (biotinylation) on tumor penetration and intracellular uptake into cancer cells could depend on the protein surface charge.

**Fig. 2:**
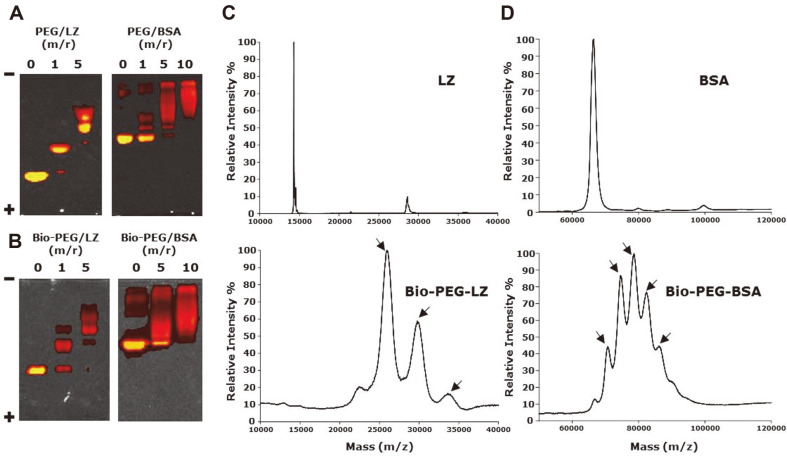
Characterization of biotinylated and PEGylated proteins. (**A, B**) Polyacrylamide gel electrophoresis of (**A**) PEG-LZ and PEG-BSA and (**B**) Bio-PEG-LZ and Bio-PEG-BSA at different PEG/protein molar ratios. (**C, D**) MS of (**C**) LZ and Bio-PEG-LZ and (**D**) BSA and Bio-PEG-BSA.

After the preparation of PEG-LZ and PEG-BSA at different PEG/protein molar ratios, gel migration of PEG-LZ and PEG-BSA was observed as compared to that of LZ and BSA, respectively (Fig. 2A). The location of most PEG-LZ at a PEG/LZ molar ratio of 1 (PEG-LZ 1) was greatly shifted as compared to that of LZ. Moreover, unmodified BSA was still observed in PEG-BSA at a PEG/BSA molar ratio of 1 (PEG-BSA 1). However, most of the BSA was successfully conjugated with PEG at a PEG/BSA molar ratio of 5 (PEG-BSA 5). According to the Image J program analysis, 89.1 ± 9.9 and 99.5 ± 0.1% LZ were conjugated with PEG at PEG/LZ molar ratios of 1 and 5, respectively. In addition, 51.5 ± 13.0, 97.8 ± 0.1, and 99.4 ± 0.6% BSA were conjugated with PEG at PEG/BSA molar ratios of 1, 5, and 10, respectively. Fig. 2B shows the gel migration of Bio-PEG-LZ (molar ratios of 0, 1, and 5) and biotin-PEG-BSA (molar ratios of 0, 5, and 10) after incubation in a PBS solution overnight. Most Bio-PEG-LZ and Bio-PEG-BSA showed significantly retarded migration as compared with that of unmodified LZ and BSA, respectively. According to the Image J program analysis, 70.9 ± 1.3 and 98.4 ± 1.9% LZ were conjugated with Bio-PEG at Bio-PEG/LZ molar ratios of 1 and 5, respectively. In addition, 80.2 ± 0.4 and 95.6 ± 5.5% BSA were conjugated with Bio-PEG at Bio-PEG/BSA molar ratios of 5 and 10, respectively. To confirm the conjugation of PEG and biotin to LZ and BSA, the molecular weights of the conjugates (Bio-PEG-LZ 5 and Bio-PEG-BSA 10) were assessed using MS (Figs. 2C and 2D). In Fig. 2C, LZ was observed at a mass of 14304 while peaks of Bio-PEG-LZ (arrows in Fig. 2C bottom panel) were identified at masses of 26123, 29641, and 33989, which corresponds to tri-, tetra-, and penta- Bio-PEG conjugated LZ. Fig. 2D shows five peaks for Bio-PEG-BSA, as indicated by the arrows. Mono-, di-, tri-, tetra-, and penta-PEGylated BSA had molecular weights of 70881, 74754, 78575, and 82297, respectively, compared to free BSA with a weight of 66948. Gel electrophoresis and MALDI-TOF analysis revealed that PEG and biotin were successfully conjugated to LZ and BSA, respectively.

### Cellular Uptake of Proteins Conjugates

**Fig. 3:**
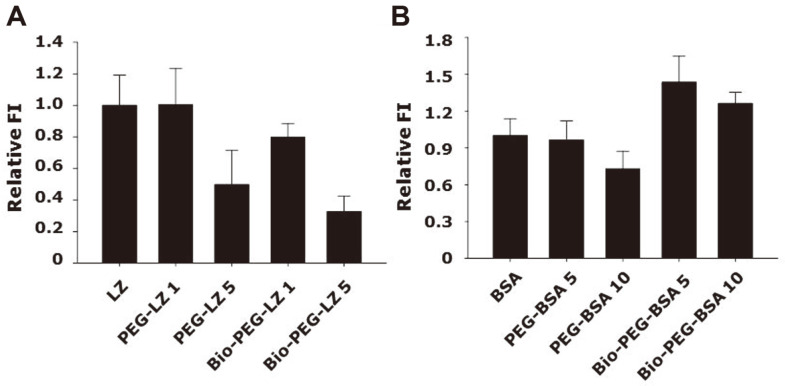
Intracellular uptake of FITC-labeled protein conjugates for A549 cells. Levels of fluorescence intensity within cells after treatment with (**A**) FITC-labeled LZ conjugates and (**B**) FITC-labeled BSA conjugates. Data are expressed as mean ± standard deviation.

Intracellular uptake of FITC labeled LZ conjugates in A549 cells was quantitatively examined after treatment with five types of samples (LZ, PEG-LZ 1, PEG-LZ 5, Bio-PEG-LZ 1, and Bio-PEG-LZ 5) for A549 cells in a 2D culture for 4 h. As shown in Fig. 3A, PEGylation of LZ attenuated the extent of intracellular uptake as compared with that of unmodified LZ. In addition, targeting ligand conjugation (biotinylation) of LZ also showed negative effects on the intracellular uptake of LZ. The relative fluorescence intensities of LZ, PEG-LZ 5, and Bio-PEG-LZ 5 within cells were 1.0 ± 0.2, 0.5 ± 0.2, and 0.3 ± 0.1, respectively. PEG-LZ 5 and Bio-PEG-LZ 5 showed similar reductions in intracellular uptake, which were not statistically significant. These results indicated that targeting ligand conjugation of cationic proteins showed negligible effects on intracellular uptake in 2D culture conditions. Fig. 3B shows the intracellular uptake of PEGylated and biotinylated BSA. PEGylated BSA significantly reduced intracellular uptake as compared with unmodified BSA. However, the extent of intracellular bio-PEG-BSA was significantly higher than that of unmodified BSA. The relative fluorescence intensities of BSA, PEG-BSA 10, and Bio-PEG-BSA 10 were 1.0 ± 0.1, 0.7 ± 0.1, and 1.3 ± 0.1, respectively. Bio-PEG-BSA 5 exhibited significantly higher intracellular uptake than that of PEG-BSA 5. This result suggested that the biotinylation of anionic proteins significantly improved intracellular uptake as compared to that of unmodified BSA and PEG-BSA. In addition, PEG-BSA 5 and PEG-BSA 10 showed significantly reduced intracellular uptake, consistent with PEGylated LZ. Taken together, biotinylation of an anionic BSA protein improved intracellular uptake, whereas biotinylation of cationic LZ elicited negligible or negative effects on cellular uptake. Cationic proteins favorably interacted with the plasma membrane owing to charge interactions, while limited interaction of anionic proteins could occur with the plasma membrane owing to charge repulsion [23-25]. In addition, reduction of the protein charge in Bio-PEG-LZ by modification of primary amine groups during conjugation could also affect the low intracellular uptake of Bio-PEG-LZ.

### Spheroid Penetration of Proteins Conjugates

**Fig. 4:**
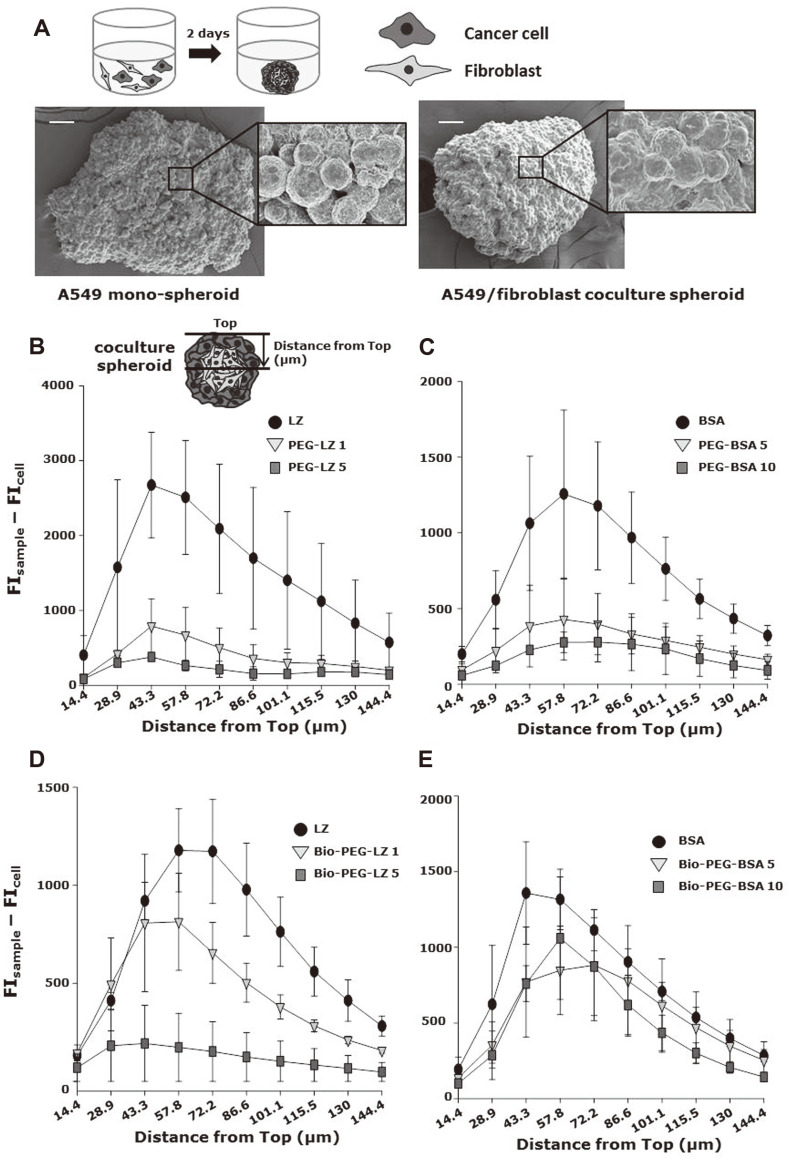
Spheroid penetration assay. (**A**) SEM images of A549 monospheroid and A549/fibroblasts coculture spheroids. (**B, D**) Quantitative analysis of spheroid penetration of (**B**) FITC-labeled LZ and PEG-LZ, (**C**) FITC-labeled BSA and PEG-BSA, (**D**) FITC-labeled LZ and Bio-PEG-LZ, and (**E**) FITC-labeled BSA and Bio-PEG-BSA at a different distance from the top for A549/fibroblast coculture spheroids.

To examine the tissue penetration of LZ and BSA conjugates, two kinds of tumor spheroids, A549 spheroid and A549/fibroblasts coculture spheroid, were prepared and visualized using scanning electron microscopy (SEM), as shown in Fig. 4A. A549 spheroids were flattened aggregated, whereas A549/fibroblasts coculture spheroid had a 3D spherical shape. The diameters of A549 spheroids and A549/fibroblasts coculture spheroids were 413.3 ± 36.8 and 333.8 ± 24.8 μm, respectively. The inset image in Fig. 4A shows the association between individual cancer cells within the spheroids. In a previous study, monoculture spheroids formed irregular and loose aggregates, whereas coculture spheroids provided compact and spherical aggregates, which was consistent with our study [26]. Accordingly, A549/fibroblasts coculture spheroids were harnessed for spheroid penetration assay. Figs. 4B-4E shows the spheroid penetration assay of FITC-labeled cationic LZ conjugates and BSA conjugates for coculture spheroids. As shown inset image of Fig. 4B, fluorescence intensities at different distances from the top of spheroids were quantitatively examined using confocal microscopy. PEG-LZ exhibited significantly decreased spheroid penetration as compared with that of unmodified LZ (Fig. 4B). The fluorescence intensities of PEG-LZ at PEG/LZ molar ratios of 0, 1, and 5 were 2508.7 ± 760.8, 669.8 ± 368.7, and 262.4 ± 70.3 at a distance of 57.8 μm from the top (Fig. 4B), respectively. Fig. 4C shows the spheroid penetration of anionic protein PEG-BSA conjugates. The fluorescence intensity of PEG-BSA at PEG/BSA molar ratios of 0, 5, and 10 were 1257.2 ± 554.5, 427.6 ± 266.8, and 276.1 ± 67.5 at a distance of 57.8 μm from the top, respectively. These results suggested that PEGylation could disturb tissue penetration of both cationic and anionic proteins. Tissue penetrations of biotinylated cationic and anionic proteins were also examined, as shown in Figs. 4D-4E. Fig. 4D shows that the degree of spheroid penetration of LZ and Bio-PEG-LZ at a PEG/LZ molar ratio of 1 was not significantly different, whereas Bio-PEG-LZ 5 showed reduced tissue penetration. The fluorescence intensities of Bio-PEG-LZ at PEG/LZ molar ratios of 0, 1, and 5 were 1178.1 ± 211.5, 814.0 ± 247.5, and 173.7 ± 44.9 at a distance of 57.8 μm from the top, respectively. Fig. 4E shows the tissue penetration of the anionic protein BSA and Bio-PEG-BSA conjugates. The differences in the degree of spheroid penetration of the Bio-PEG-BSA conjugates and unmodified BSA were not significant. The fluorescence intensities of Bio-PEG-BSA at PEG/BSA molar ratios of 0, 5, and 10 were 1316.7 ± 200.2, 848.1 ± 293.0, and 1060.1 ± 405.1 at a distance of 57.8 μm from the top, respectively. These results indicated that the conjugation of targeting ligand to anionic proteins could not interfere with tissue penetration. In addition, considering Bio-PEG-LZ 1 exhibited better tissue penetration than PEG-LZ 1, biotinylation on cationic protein could slightly recover the decreased extent of tissue penetration by PEGylated protein in case of a low degree of modification. In a previous study, antibodies with low affinity penetrated deeper than those with high affinity because of their low interaction with target cells and bypass [27]. It was likely that the interaction between the plasma membrane and biotinylated cationic protein (Bio-PEG-LZ) was too strong to penetrate tissues, whereas that between cells and biotinylated anionic protein was appropriate for tissue penetration. In addition, these results indicated that PEGylation of anionic proteins significantly interfered with intracellular uptake and tissue penetration, whereas targeting ligand conjugation to anionic proteins could provide enhanced intracellular uptake without a significant decrease in tissue penetration. However, the PEGylation and biotinylation of cationic proteins could result in insignificant intracellular uptake and decreased tissue penetration.

### Spheroid Penetration Assay of Cit -LZ

**Fig. 5:**
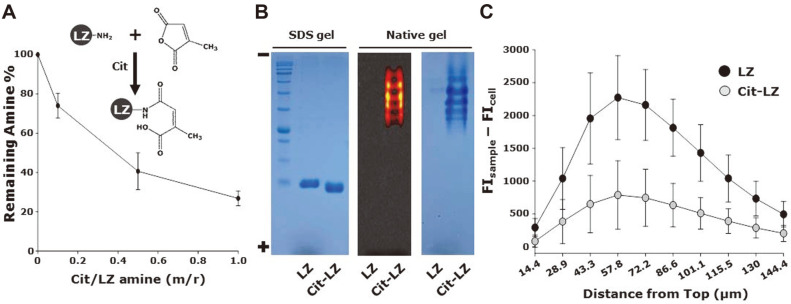
Effect of citraconylation on spheroid penetration of proteins. (**A**) Relative extent of remaining primary amine groups in LZ after a citraconylation reaction. (**B**) Gel electrophoresis of LZ before and after citraconylation using native polyacrylamide gels and SDS (%)-containing polyacrylamide gels. (**C**) Fluorescence intensities of FITC-labeled LZ and Cit-LZ at different distances from the top for GFP-expressing A549/fibroblast coculture spheroids.

In this study, the primary amine groups of proteins were modified with PEG, which resulted in changes in the number of primary amine groups in the proteins. To examine the effects of amine modification on the degree of tissue penetration of proteins, primary amine groups were modified with citraconic anhydride according to a previous study [28]. Fig. 5A shows the number of amine groups remaining after the reaction of LZ with Cit in Cit/amine groups of LZ molar ratios of 0, 0.1, 0.5, and 1. As the Cit/amine groups of the LZ molar ratios increased, the number of remaining amine groups decreased. The remaining amine groups were 26.8 ± 3.8% in Cit/amine groups with LZ molar ratios of 1. After preparation of Cit-LZ in Cit/amine groups of LZ molar ratios of 1, migrated FITC labeled Cit-LZ and LZ were visualized by fluorescence imaging and Coomassie staining after acrylamide gel electrophoresis, respectively (Fig. 5B). LZ and Cit-LZ were observed in SDS-containing polyacrylamide gels (Fig. 5B, left panel). Cit-LZ was observed in the fluorescence images and Coomassie staining in the absence of SDS owing to charge change by citraconylation. However, owing to the positive charge of LZ, migration of LZ was not observed in the absence of SDS in native gels. The ladder-like band migration of Cit-LZ in the fluorescence image (Fig. 5B, middle panel) and Coomassie staining image (Fig. 5B, right panel) could be attributed to different citraconylation degrees of LZ. Fig. 5C shows the spheroid penetration of FITC labeled LZ and Cit-LZ in A594/fibroblast coculture spheroids. After citraconlyation, the degree of spheroid penetration noticeably decreased. The fluorescence intensities of LZ and Cit-LZ were 2272.2 ± 639.4 and 789.2 ± 521.3 at a distance of 57.8 μm from the top, respectively (Fig. 5C). Cit-LZ showed significantly reduced spheroid penetration as compared with that of LZ, probably owing to differences in charge, indicating that the cationic charge of the protein played a pivotal role in spheroid penetration. In a recent study, cationic nanomaterials showed a much deeper intratumor penetration than anionic nanomaterials, which was consistent with our study [29]. In addition, these results suggested that amine modification during PEGylation could be one of the crucial causes of reduced tissue penetration in PEGylated proteins.

In this study, cationic LZ and anionic BSA were conjugated with PEG and biotin-PEG, respectively, to examine the effects of chemical conjugation on intracellular uptake and spheroid penetration of protein conjugates. PEGylation and biotinylation of cationic LZ resulted in decreased intracellular uptake for A549 cells. However, biotinylation of anionic BSA allowed elevated intracellular uptake, whereas PEGylation interfered with the intracellular uptake of BSA conjugates as compared with unmodified BSA. PEG-LZ and PEG-BSA showed much lower spheroid penetration than that of unmodified LZ and BSA, respectively, indicating that PEGylation resulted in the decrease in tissue penetration regardless of the protein charge. However, Bio-PEG-BSA showed comparable tissue penetration to that of unmodified BSA, whereas Bio-PEG-LZ exhibited significantly reduced tissue penetration. In addition, Cit-LZ showed reduced spheroid penetration compared to that of LZ, probably owing to the reduced net charge of proteins. In this study, we demonstrated that the effects of PEGylation and biotinylation on tissue penetration and intracellular uptake depended on the net charge of the proteins. Thus, targeting ligand-PEG conjugation to anionic proteins could be an excellent strategy to improve their cytosolic delivery to target cancer cells and in vivo penetration, whereas modification of cationic proteins should be designed differently to achieve better results.
